# Analysis of KRT1, KRT10, KRT19, TP53 and MMP9 expression in pediatric and adult cholesteatoma

**DOI:** 10.1371/journal.pone.0200840

**Published:** 2018-07-18

**Authors:** Enikő Palkó, Szilárd Póliska, István Sziklai, András Penyige

**Affiliations:** 1 Department of Human Genetics, Faculty of Medicine, University of Debrecen, Debrecen, Hungary; 2 Genomic Medicine and Bioinformatics Core Facility, Department of Biochemistry and Molecular Biology, Faculty of Medicine, University of Debrecen, Debrecen, Hungary; 3 Department of Otorhinolaryngology, Head and Neck Surgery, Faculty of Medicine, University of Debrecen, Debrecen, Hungary; 4 Faculty of Pharmacy, University of Debrecen, Debrecen, Hungary; University of South Alabama Mitchell Cancer Institute, UNITED STATES

## Abstract

Cholesteatoma is an epidermal cyst with still unknown pathomechanism. The aim of the current study was to investigate molecular differences in the background of the hyperproliferative property and aggressive behavior typical of the cholesteatoma epithelium. The expression of three cytokeratin genes (KRT1, KRT10 and KRT19), the matrix metalloproteinase 9 gene (MMP9) and the tumor suppressor TP53 gene was measured by qRT-PCR in surgical samples of pediatric and adult cholesteatoma cases and their expression level was compared to that of normal skin samples from the retroauricular region of control individuals. Cholesteatoma samples were stratified according to the age of onset and recurrence for more detailed analysis. Our results showed identical expression pattern for KRT1 and KRT10, their expression was higher in pediatric cases than in adults, especially in pediatric recurrent samples. The expression level of KRT19 was inversely proportional to that of KRT1/KRT10, it was lower in the more invasive recurrent cases both in our pediatric and adult groups. As it was expected from the bone destructive behavior of cholesteatoma, a significantly elevated expression of MMP9 was measured in cholesteatoma samples, the highest level was found in adult recurrent cases. Low expression levels characterize the TP53 gene without significant differences in our samples. These findings demonstrate that cytokeratin expression distinguishes between pediatric/adult, nonrecurrent/recurrent cases, suggesting that distinct differentiation state and cell division potential characterize these cholesteatoma cases. KRT19 with a tumor suppressor potential might restrict the recurrence of cholesteatoma. The differences observed in gene expression profiles between cholesteatoma and control samples support the notion that cholesteatoma is a cystic lesion with tumor-like behavior because it is characterized by invasive, destructive growth and high tendency for recurrence.

## Introduction

Aural cholesteatoma is a consequence of chronic middle ear inflammation. Cholesteatoma—previously called as pearl tumor due to its characteristic pocket-like appearance—itself is a keratinous cystic lesion localized in an ectopic position in the middle ear. Histologicaly the epithelial pocket of cholesteatoma is an epidermal cyst enclosed by multilayered keratinized epithelium. Since keratin produced by the keratinized epithelium accumulates inside the pocket which cannot be emptied, the size of the pocket is continuously growing. The locally invasive nature of cholesteatoma—its proliferation and growth—affects various parts of the middle ear to varying degrees, frequently leading to expansive ossicular chain destruction, hearing loss, otorrhea, dizziness, and intracranial complications. To date, cholesteatoma is a microsurgical challenge for the ear, nose and throat specialists. Recurrence is common even after successful surgical treatment, especially in childhood cases [1–2].

Despite of the large number of studies the precise ethiopathogenesis of cholesteatoma is still not clarified, however, numerous theories about the formation of cholesteatoma had been presented in the literature. Based on these studies in terms of etiology cholesteatoma could be classified into two types: acquired and the congenital.

The congenital type is a developmental disorder, during tissue migration in the temporal region separated keratinized squamous epithelial cells or islands became trapped into an abnormal mesodermic region behind the intact tympanic membrane during embryogenesis. Conventionally six basic theories are suggested about the pathogenesis of the acquired cholesteatoma and the formation of epithelial pockets [1–5].

Since the origin of cholesteatoma is still disputed numerous molecular studies have been carried out to find molecular defects that might explain its etiopathogenesis. The presence of various growth factors, inflammatory regulatory mediators, HPV viruses, factors responsible for bone destruction, angiogenesis and differentiation had been detected in cholesteatoma, all these factors may play a role in the formation and aggressive behavior of cholesteatoma [6–9].

Interestingly, a few recent publications suggested the possibility, that based on its invasive and destructive spread and frequent recurrence, cholesteatoma is a keratinous cyst behaving like a tumor. Results of the latest cytogenetic research might moderately support this idea since several research groups have found chromosomal abnormalities in cholesteatoma samples. Numerical abnormalities of Chr7, Chr8 and Chr17 were detected [10–12]. However, according to our knowledge only a single case of malignant transformation of cholesteatoma was reported in the literature [13].

Beside the cytogenetic studies, newly developed high throughput molecular methods were also applied to recognize differently expressed genes and proteins in cholesteatoma samples. Using DNA microarray a gene expression pattern somewhat similar to tumor samples was recognized. Several tumor relevant genes, numerous differently expressed genes coding for interleukins, cytokeratins responsible for the induced inflammatory pathways seen in cholesteatoma, and protein degrading enzymes were detected [14–16]. The observed gene expression patterns might explain the aggressive behavior and local destruction caused by the development of cholesteatoma.

Overexpression of proteins involved in inflammation, response to bacteria, and/or protein degradation and participating in cancer pathways were found in a large scale proteomic study of cholesteatoma. The same study showed a keratin expression pattern suggesting lower grade of cell differentiation in cholesteatoma [17].

The purpose of our study was to investigate the mRNA expression patterns of five selected genes—KRT1, KRT10, KRT19, MMP9, and TP53—which might have an influence on the pathogenesis of cholesteatoma, and to identify differences in the multiple gene expression profiles in cholesteatoma and control skin samples. Another aim was to validate some of the findings of the high throughput studies in a smaller group of patients.

## Materials and methods

### Sample collection

The study was approved by the Research Ethics Committee of University of Debrecen Medical and Health Science Center (protocol number 3047–2009). All participating patients were properly informed and a signed written consent was obtained from adult patients and from parents of minors or children before recruitment. The study was conducted in compliance with the principles of the Declaration of Helsinki (1964). As it was described previously 26 patients with acquired cholesteatoma comprised of 11 females and 15 males were recruited in the study [18]. The age of cholesteatoma patients ranged between 4 and 65 years (average: 23.4 years). The tympanic membrane was perforated in all cholesteatoma patients, and all patients underwent primary or secondary surgery. Patients were divided into a pediatric (15 cases; 0–18 years) and an adult group (11 cases; over 19 years). The diagnosis of cholesteatoma in all specimens was confirmed by histopathological examination. Demographic data and a brief clinical history including the age, gender of the patients and surgical parameters of cholesteatoma were described in [18]. In addition to the 26 cholesteatoma samples, 5 control skin samples from the retroauricular region of healthy individuals were also obtained.

### Total RNA extraction

To analyze gene expression levels, total RNA content was extracted from surgical samples collected immediately following surgery. Samples were fixed in RNA*later* RNA Stabilization Reagent (Life Technologies) (approximately 50 μL/mg tissue) and stored at 4°C until RNA extraction. After removing the excess RNA*later* solution from samples the matrixes of cholesteatoma specimens were manually cleaned from the surrounding tissues. As an average, 70 mg tissue sample were cut into thin slices and homogenized manually in TRI Reagent (Molecular Research Center INC, Cincinnati, USA) using a glass-Teflon tissue homogenizer. The RiboPure kit [Ambion (Europe) LTD, Huntingdon, UK] was used to extract total RNA from the homogenates according to the manufacturer’s instructions. RNA concentration, quality and integrity were evaluated by NanoDropTM 1000A spectrophotometer (Thermo Fisher Scientific, Woolston, UK). The average RNA yield was 0.4 μg/μL and the purity index (ratio 260/280 and 260/230) was above 1.85 in all samples.

### Reverse transcription

To generate first-strand cDNA a total of 2 μg of RNA was reverse transcribed in a 20  μL reaction volume using the High Capacity cDNA Kit with RNase inhibitor (Thermo Fisher Scientific, Woolston, UK). Briefly, 2 μg total RNA was mixed with 10.0 μL of 2× reverse transcriptase (RT) buffer, 1.0 μL 20× enzyme mix and nuclease-free water to a total volume of 20 μL. The reaction mix was then incubated first at 25°C for 10 min then at 37°C for 120 min; the reaction was terminated by incubation at 95°C for 5 min and then chilling them immediately on ice for an additional 5 min.

### Primer and probes and real-time PCR detection

Target sequences were quantitated by TaqMan methodology based quantitative real-time PCR (RT-PCR). The custom made, gene specific pre-validated TaqMan Gene Expression Assays (Assays-On Demand IDs: TP53: Hs00153408_m1; KRT1: Hs00196158_m1; KRT10: Hs00166289_m1; KRT19: Hs00761767_m1; MMP9: Hs00957562_m1) and the TaqMan Gene Expression Master Mix containing AmpliTaq Gold® DNA Polymerase were purchased from Thermo Fisher Scientific, Woolston, UK. Gene expression measurements were carried out with an ABI Prism 7900HT Sequence Detection System (Thermo Fisher Scientific, Woolston, UK) according to the manufacturer’s instructions; briefly 4 ng of cDNA was diluted into a total of 20 μL reaction volume containing 10 μL TaqMan Fast Universal PCR Master Mix (Thermo Fisher Scientific) with AmpliTaq Gold DNA Polymerase and the target gene specific TaqMan Gene Expression Assay mix. Amplification was performed for 40 cycles, including denaturation at 95°C for 15 seconds, annealing at 60°C and extension at 72°C for 60 and 30 seconds, respectively. Relative quantification of mRNA expression levels of the target genes was performed using the comparative threshold method using the PPIA gene as an endogenous reference [Assays-On Demand ID: Hs99999904_m1 (Thermo Fisher Scientific)].

### Statistical analysis

Results were statistically analyzed using GraphPad Prism 5.0 (GraphPad Software, Inc.; San Diego, CA, USA). Descriptive column statistics of each data set were performed and the distribution of data was analyzed by Kolmogorov-Smirnov test. To assess the statistical significance of differences in gene expression between multiple groups the nonparametric one-way ANOVA Kruskal–Wallis test (K-W test) in combination with the *post hoc* Dunn’s test to adjust for multiple comparisons was applied. In all tests difference was considered significant as p<0.05. Dunn’s p values were indicated as: p < 0.05(*); p < 0.01(**). The magnitude of the effect of a given gene was determined by calculating the effect size, the Cohen’s *d* value.

## Results

Demographic data and surgical parameters of the 26 acquired cholesteatoma patients (comprising of 15 children and 11 adults) recruited into our study were described previously [18]. To broaden our analysis, patients were divided into two groups according to their age: a pediatric (younger than 18 years old) and an adult (above 18 years) group then both groups were further classified based on the clinical data into primary acquired (nonrecurrent) and recurrent groups. Analyzing the extent of cholesteatoma, we have found that smaller regions of the middle ear were affected in the nonrecurrent cases compared to the recurrent cases, the extension of cholesteatoma to more than 2 anatomical regions was more common in recurrent samples. Based on the clinical data, the extent of bone destruction was also greater in recurrent cases than in nonrecurrent samples. Out of the examined 26 cases of acquired cholesteatoma, the auditory ossicular chain was destroyed in 24 patients, sound ossicular chain was observed only in 2 cases. The recurrence was more common in childhood cholesteatoma than in adult cases.

**Fig 1:**
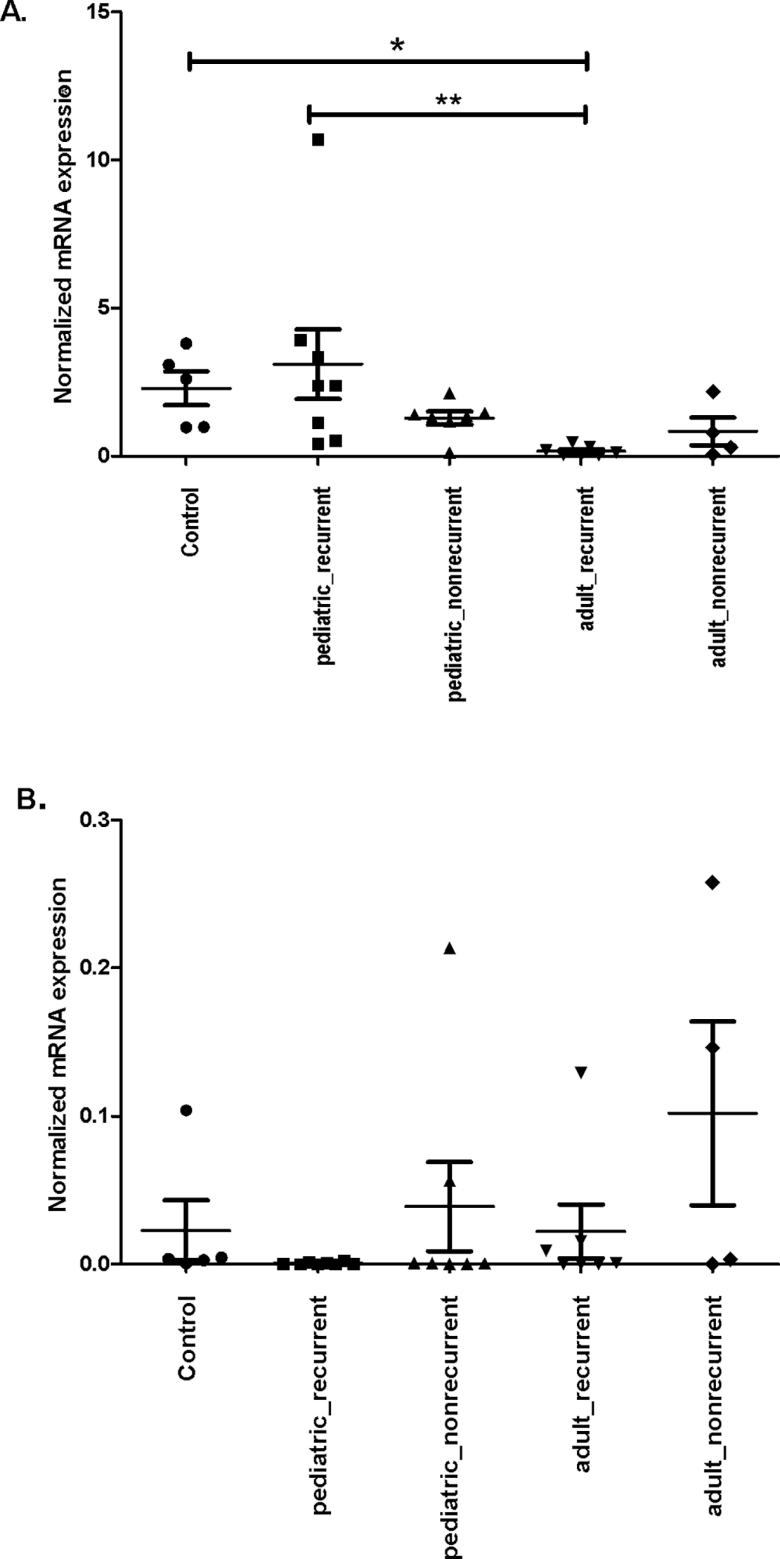
Expression of KRT10 and KRT19 genes in the stratified cholesteatoma patient groups and control samples. Surgical samples were homogenized, total RNA was prepared and the cDNA generated was used as template for quantitative RT-PCR. Expression values were normalized to the PPIA gene. (A) The expression of KRT10 is significantly lower in adult recurrent samples than in pediatric recurrent cases or control samples. B. No significant difference was found in KRT19 expression between control and cholesteatoma recurrent or nonrecurrent samples. The mean and standard error of the mean values are shown. The significance levels are: (*) P-value < 0.05, (**) P-value < 0.01. The mean and standard error of the mean values are shown.

To investigate the expression of target genes, the normalized mRNA levels measured in cholesteatoma samples were compared to those of normal skin samples of control individuals.Although the KRT1 and KRT10 genes located on different chromosomes (KRT1: 12q12-q13; KRT10: 17q21) they are expressed together during the terminal differentiation of keratinocytes in a coordinated fashion, their protein products form functional dimers during IF formation. According to our expectation, the PPIA normalized expression of two genes showed identical expression patterns, however, the expression level of KRT10 was higher than that of KRT1 (data not shown). The assessment of our RT-PCR data showed that mRNA expression was significantly different between the stratified groups (Kruskal-Wallis (K-W) *P =* 0.004 and K-W P = 0.0031 for KRT1 and KRT10, respectively). In samples of the pediatric recurrent group KRT10 expression level was significantly higher compared to that of the adult recurrent group (μ±SD = 3.106 ± 3.150 and μ±SD = 0,172 ± 0.166, respectively; the K-W test followed by Dunn’s P< 0.01). The effect size of KRT10 gene in this comparison is 1.27. KRT10 mRNA expression was also significantly higher in the control samples compared to that of the adult recurrent group (μ±SD = 2.287 ± 1.270 and μ±SD = 0,172 ± 0.166, respectively; the K-W test followed by Dunn’s P< 0.05), (Fig 1A). The effect size of KRT10 is -2.33.

The difference in KRT19 expression was not significantly different between control, recurrent and nonrecurrent samples, the lowest expression level was observed in samples of the two recurrent groups (μ±SD = 6.750 × 10^−4^ ± 7.730 × 10^−4^ and μ±SD = 2.209 × 10^−2^ ± 0.047, in the pediatric and adult groups, respectively) (Fig 1B).

**Fig 2:**
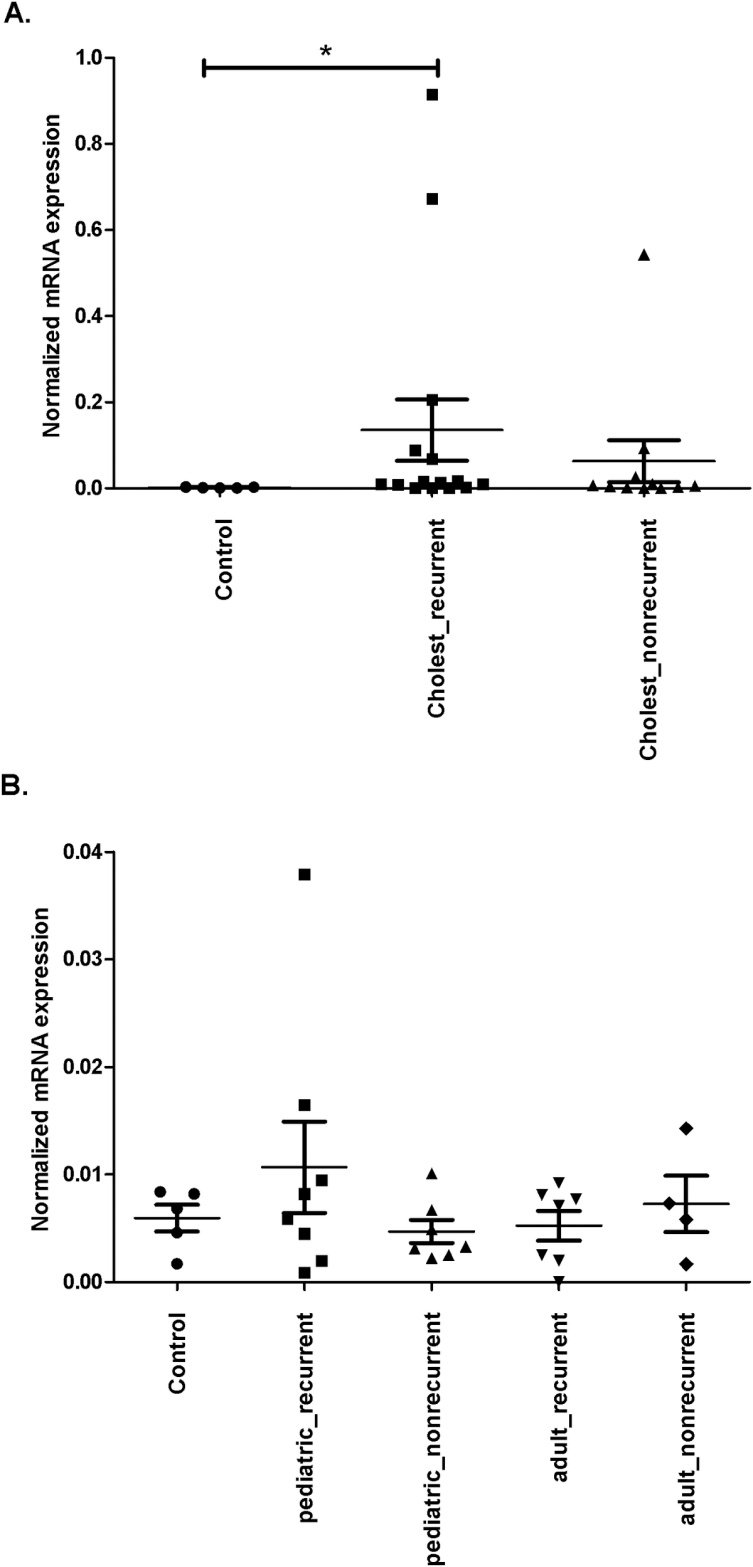
MMP9 and TP53 mRNA expression profiles in stratified cholesteatoma and control samples. Surgical samples were homogenized, total RNA was prepared and the cDNA generated was used as template for quantitative RT-PCR. Expression values were normalized to the PPIA gene. (A) Significantly elevated MMP9 expression is seen in recurrent cholesteatoma samples compared to nonrecurrent cholesteatoma samples or control skin samples. (B) There is no significant difference in TP53 expression levels among the groups. The mean and standard error of the mean values are shown. The significance level is (*) P-value < 0.05. The mean and standard error of the mean values are shown.

In case of MMP9 mRNA expression was significantly elevated in recurrent cholesteatoma samples compared to the normal skin (μ±SD = 1.351 × 10^−1^ ± 0.276 for recurrent cases and μ±SD = 1.046 × 10^−1^ ± 0.2336 for control samples; the K-W test followed by Dunn’s P< 0.05) (Fig 2A). The effect size of MMP9 gene is 0.90. Following stratification significant differences in MMP9 mRNA expression was no longer observed between all age groups and control samples. The highest MMP9 expression was measured in adult and pediatric recurrent samples, the lowest was observed in the adult nonrecurrent group (μ±SD = 1.517 × 10^−1^ ± 2.401 × 10^−1^, μ±SD = 1.205 × 10^−1^ ± 3.211 × 10^−1^ and μ±SD = 4.022 × 10^−3^ ± 4.379 × 10^−3^, respectively).

The expression of the TP53 gene was higher in the recurrent cases (μ±SD = 8.132 × 10^−3^ ± 9.274 × 10^−3^) than in nonrecurrent ones (μ±SD = 5.632 × 10^−3^ ± 2.808 × 10^−3^). The highest TP53 expression after stratification was found in samples of children with recurrent cholesteatoma (μ±SD = 1.068 × 10^−2^ ± 1.204 × 10^−2^) and a somewhat lower level was observed in the adult nonrecurrent group (μ±SD = 7.269 × 10^−3^ ± 5.256 × 10^−3^). However, mRNA expression in the stratified groups was not significantly different from one another (Fig 2B).

We have to note, that some of our data sets contain outliers (especially KRT19 and MMP9), and those can affect the outcome of tests. We did not want to reduce the number of data points by trimming our data, especially since outliers are not necessarily bad data, they may represent variability in the measurement indicating skewness in the distribution of our expression values.

**Fig 3:**
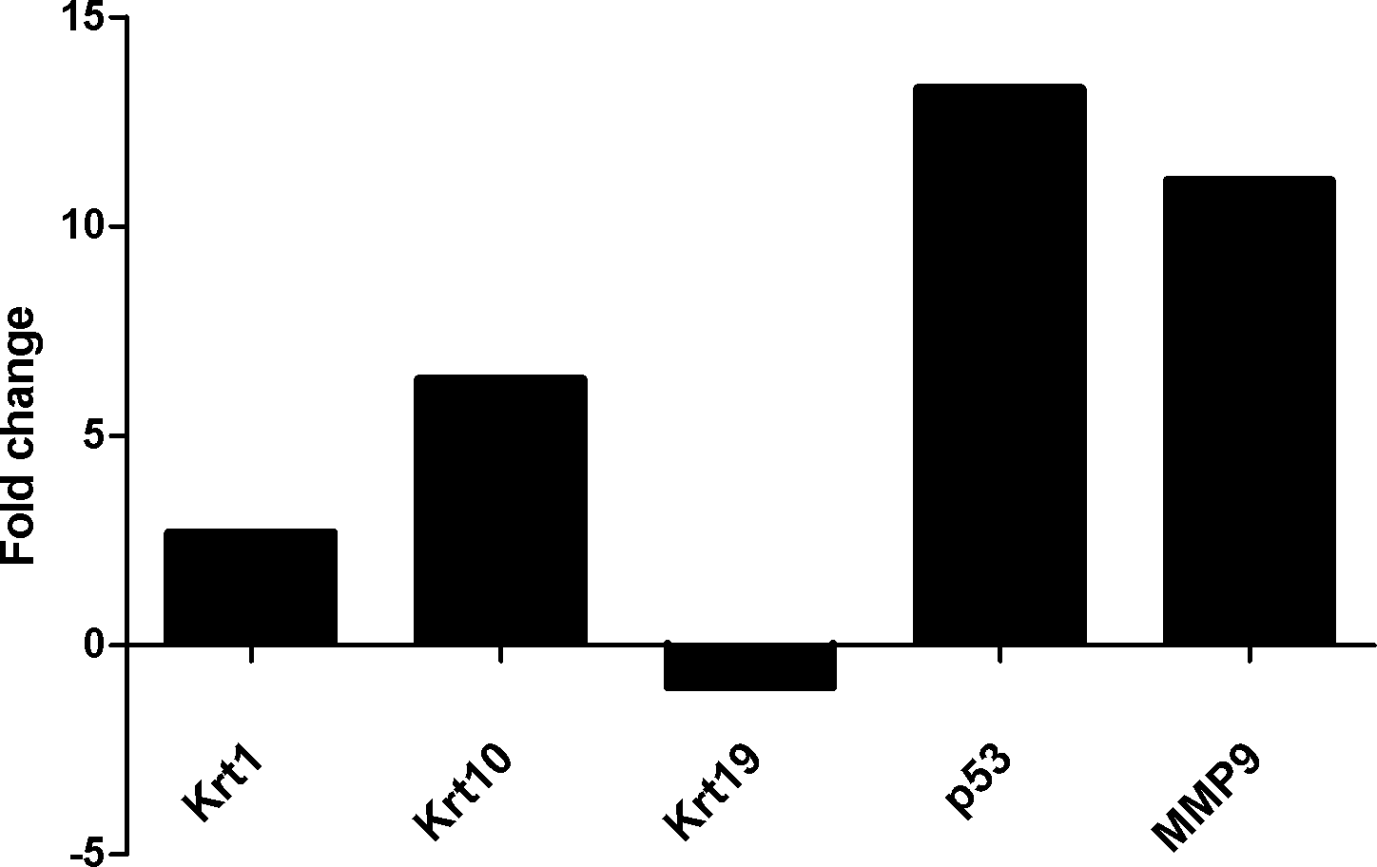
Fold changes in the expression of KRT1, KRT10, KRT19, MMP9, TP53 in samples of a patient showing cholesteatoma recurrence during the study period. To calculate the fold change the difference between the expression values of the recurrent and the primary samples was divided by the expression value of the primer sample.

During the sample collection period of our study a pediatric patient developed recurrent cholesteatoma. It gave us a chance to compare expression levels of all the studied genes between the primer and recurrent cholesteatoma samples of the same person. The calculated fold changes are shown in (Fig 3), all genes had an elevated expression level in the recurrent sample with the exception of KRT19 which had a much reduced expression.

## Discussion

Cholesteatoma suppurative otitis media is the result of a chronic inflammatory process. Histologicaly cholesteatoma itself is a cyst enclosed in a multi-layered keratinizing squamous epithelium wall, containing epithelial keratin debris. It is characterized by locally invasive behavior and frequent recurrence due to uncoordinated cell proliferation and extensive bone destruction. The suggested pathomechanism of cholesteatoma involves an inflammatory process associated with changes in cell signaling pathways and possibly genetic defects. The characteristic aggressive behavior of cholesteatoma might be the result of interactions between these factors [2].

Previously we have reported a significantly elevated c-MYC expression level in cholesteatoma samples, now we have determined the mRNA expression pattern of five additional genes in samples of our original cohort to obtain additional information about the molecular background of cholesteatoma. In this study patient samples were stratified into four groups according to age of onset and clinicopathologic characteristics of patients: pediatric (age below 18 year) recurrent or nonrecurrent cholesteatoma and adult (age above 18 year) recurrent or nonrecurrent cholesteatoma. The quantitative assessment of the expression KRT1, KRT10, KRT19, MMP9 and TP53 genes was carried out in our surgical samples using RT-QPCR methodology. Normal skin samples derived from the retroauricular region of healthy individuals were used as control for all measurements.

First we have assessed the expression pattern of the cytokeratin genes KRT1, KRT10 and KRT19. It was warranted by previous studies which have suggested the involvement of a number of cytokeratins in the background of the hyperproliferative phenotype of cholesteatoma [19–23]. Cytokeratins are members of the IF superfamily present in cells with epithelial origin, typically expressed in a highly regulated, tissue-specific, cell cycle stage and differentiation-dependent manner. Therefore cytokeratins frequently used as markers of cell migration, differentiation and proliferation state in epithelial cells. Moreover recent reports suggest that interactions between keratins and other proteins modulate signaling cascades that regulate cell migration, invasion, and metastasis. According to this suggestion several cytokeratins were found differently expressed in malignant epithelial cells, their specific expression pattern frequently correlated with the prognosis and metastatic potential of certain tumors, like in breast cancer and tumors of the oral cavity [24–25]. KRT1 is coexpressed with KRT10 in the suprabasal layer of the epidermis, elevated expression of the KRT1/KRT10 complex indicate increased terminal differentiation of keratinocytes. KRT19 is specifically expressed in the periderm of the developing epidermis [26]. Although KRT19 is one of the most frequently used marker for tumor cell detection, recent results showed, that KRT19 is a potential tumor suppressor. It negatively regulates Akt signaling through modulation of Egr1 nuclear localization. It also down regulates cell proliferation and migration through promoting the nuclear translocation of β-catenin thus modulating the NUMB-dependent NOTCH signaling pathway [22], [27–29]. In normal skin, the epithelial differentiation and proliferation processes are well balanced, therefore differences that might be observed in the expression of KRT genes between the cholesteatoma and normal skin samples could help to characterize the cellular differentiation and proliferation state of cholesteatoma.

In our cholesteatoma samples KRT1 and KRT10 showed identical expression patterns as it was expected, since they protein products are known to form heteroduplex in cells. This data could serve as an internal control to validate the accuracy of our measurements. The expression level of KRT1 and KRT10 genes in the pediatric recurrent group was higher than that of the control and nonrecurrent pediatric groups but the difference was not significant. The expression of the two genes suggests an ongoing keratinocyte differentiation and keratinization process especially in pediatric recurrent cases. The significantly reduced expression of KRT1 and KRT10 in the adult group might shift the proliferation-differentiation balance of cells toward dedifferentiation and proliferation, especially in the recurrent group.

The expression level of KRT19 appears to be inversely proportional to that of KRT1/KRT10, it was lower in the more invasive recurrent cases in our pediatric and adult groups, too. Since its reduced level might be associated with elevated cell proliferation potential its lower expression level suggests worse prognosis for recurrence. This suggestion correlates with the high recurrence frequency found in pediatric cases.

The type IV/V collagenase matrix metallopeptidase 9 (MMP9) enzyme could degrade the extracellular matrix in normal physiological processes and under pathological conditions, too. Several studies suggested that its increased expression is required for tissue remodeling during tumor progression, needed for invasion and metastasis generation. Compared to the control sample, the expression of the MMP9 was significantly increased in the recurrent cholesteatoma group, the highest mRNA expression level was found in adult recurrent cases. The elevated production of MMP9 likely plays an important role in the proteolysis of the extracellular matrix and local bone destruction. The elevated c-MYC expression we have detected previously in cholesteatoma might contribute to the increased MMP9 expression since the MMP9 promoter harbors a c-MYC binding site [18, 30–32].

The expression of the TP53 gene did not show significant differences between patients and controls in any comparisons. Compared to control samples the highest TP53 expression was found in pediatric recurrent samples, it was also elevated, although to a lesser extent in adult nonrecurrent cases. In our previous work the highest c-MYC expression level was found in children with recurrent cholesteatoma, this elevated c-MYC level might lead to higher TP53 expression since two c-MYC binding sites are present in the promoter of TP53 [33]. Although p53 activity is regulated mainly on posttranscriptional level, the somewhat elevated p53 expression might counteract the effect of over expressed c-MYC on apoptosis and cell proliferation [34–35]. C-MYC is thought to be a key factor in tumorogenesis, however over expression of c-MYC alone does not induce tumorogenesis, p53 protein can balance its effect thorough the activation of apoptosis [36]. The expression levels of KRT19 found in our samples point toward that balancing effect, too.

Changes in the expression levels of the five genes found in our single pediatric patient with recurrence during the study period nicely correlates with the gene expression patterns described above.

Based on our results it can be concluded that the expression pattern of KRT1, KRT10 and KRT19 genes seen in our pediatric cholesteatoma samples suggests elevated immature keratinocyte differentiation and elevated proliferation potential, especially in recurrent samples. On the other hand expression pattern of these markers seen in adult samples is consistent with keratinocyte dedifferentiation and cell cycle deregulation in the recurrent group, however, the elevated KRT19 expression in the nonrecurrent group might repress the latest process. Increased MMP9 production is consistent with the aggressive, destructive phenotype of cholesteatoma. TP53 mRNA expression does not seem to play an important role in the pathogenesis of cholesteatoma.

We can conclude, that the expression pattern of the five genes in our cholesteatoma samples suggests that cholesteatoma is a noncancerous tumor with a strong potential for recurrence. The differences in the expression pattern of the KRT1 and KRT10 genes observed in our study support the hypothesis that distinctly altered cellular differentiation processes are associated with cholesteatoma formation in pediatric and adult cases. KRT19 expression might have prognostic significance, its lower level in pediatric cases could be associated with the more aggressive behavior of childhood cholesteatoma.

It is clear that our study has some limitations, such as the small sample size and the low number of genes investigated. Further studies using larger populations are needed possibly in a different cohort to replicate our results in order to clarify the molecular pathomechanism of the disease and characterize differences between pediatric and adult cholesteatoma. Another limitation is the use of retroauricular skin as control instead of using samples taken from the tympanic membrane or external ear canal. We are aware of the fact that retroauricular skin is not the most appropriate control sample for cholesteatoma, however, several studies have used this sample as control according to previous publications [10, 14, 16]. Moreover, at the end of surgical removal of cholesteatoma, it is difficult to obtain a sample from the external auditory ear canal due to the necessary reconstruction of this anatomical region, or from the tympanic membrane since it is frequently damaged in cholesteatoma.

## Supporting information

S1 Table Normalized gene expression data. (XLSX)
